# Loss of Emerin Alters Myogenic Signaling and miRNA Expression in Mouse Myogenic Progenitors

**DOI:** 10.1371/journal.pone.0037262

**Published:** 2012-05-11

**Authors:** Adam J. Koch, James M. Holaska

**Affiliations:** 1 The University of Chicago Committee on Genetics, Genomics and Systems Biology, The University of Chicago, Chicago, Illinois, United States of America; 2 Department of Medicine, Section of Cardiology, The University of Chicago, Chicago, Illinois, United States of America; 3 Committee on Developmental, Regeneration and Stem Cell Biology, The University of Chicago, Chicago, Illinois, United States of America; IRCCS-Policlinico San Donato, Italy

## Abstract

Emerin is an integral membrane protein of the inner nuclear membrane. Mutations in emerin cause X-linked Emery-Dreifuss muscular dystrophy (EDMD), a disease characterized by skeletal muscle wasting and dilated cardiomyopathy. Current evidence suggests the muscle wasting phenotype of EDMD is caused by defective myogenic progenitor cell differentiation and impaired muscle regeneration. We obtained genome-wide expression data for both mRNA and micro-RNA (miRNA) in wildtype and emerin-null mouse myogenic progenitor cells. We report here that emerin-null myogenic progenitors exhibit differential expression of multiple signaling pathway components required for normal muscle development and regeneration. Components of the Wnt, IGF-1, TGF-β, and Notch signaling pathways are misexpressed in emerin-null myogenic progenitors at both the mRNA and protein levels. We also report significant perturbations in the expression and activation of p38/Mapk14 in emerin-null myogenic progenitors, showing that perturbed expression of Wnt, IGF-1, TGF-β, and Notch signaling components disrupts normal downstream myogenic signaling in these cells. Collectively, these data support the hypothesis that emerin is essential for proper myogenic signaling in myogenic progenitors, which is necessary for myogenic differentiation and muscle regeneration.

## Introduction

The nucleus is the primary site of nucleic acid regulation, including DNA replication, RNA transcription, and the organization of active and repressed chromatin domains. Proper regulation of these processes is essential for successful lineage specification and differentiation during embryonic development and for tissue repair after injury. Thus, these processes must be tightly controlled to ensure that the appropriate repertoire of genes is expressed during specification and differentiation into particular cell types.

The nucleus is separated from the cytosol by the nuclear envelope, which is composed of two lipid bilayers: the outer nuclear membrane (ONM), which is contiguous with the endoplasmic reticulum, and the inner nuclear membrane (INM). Underlying the INM is a meshwork of type V intermediate filament proteins called lamins, which are the major scaffolding component of the nuclear lamina [1]. The INM contains greater than 70 integral INM proteins, many of which bind directly to lamins. Collectively the INM proteins and lamins are referred to as the nuclear lamina. The nuclear lamina provides the nuclear envelope with the elasticity necessary to maintain proper nuclear structure under high stress loads [2] (e.g., contracting muscle). Lamins are also required for proper localization of many INM proteins to the nuclear envelope [3].

Emerin was one of the first INM proteins to be discovered [4], [5], [6] and is a founding member of the LEM-domain family of proteins that includes Lap2β, Emerin, and MAN1 [7]. Mutations in emerin cause X-linked Emery-Dreifuss Muscular Dystrophy (EDMD), a disease characterized by skeletal muscle wasting and irregular heart rhythms. The skeletal muscle phenotypes of EDMD have been attributed to an inability to regenerate damaged muscle [8], [9]. Emerin is expressed in all differentiated cells, yet emerin loss affects only skeletal muscle, heart and tendons. Thus emerin was proposed to have roles in regulating tissue-specific gene expression or cell signaling pathways. Several groups have investigated signaling disruptions in cells containing mutations in emerin or lamin A that are associated with EDMD. ERK1/2 is upregulated in emerin-null [10] and lamin A H222P mutant mouse [11] hearts. Importantly, downstream target genes were also misregulated showing that ERK signaling was disrupted in these mice [11]. Interestingly, some phenotypes in these mice could be relieved by treatment with the ERK inhibitor PD98059 [12]. C2C12 myoblasts and HeLa cells downregulated for emerin or lamin A also had perturbed ERK signaling [12]. Skeletal muscle from EDMD patients and emerin-null mice also exhibit increased expression of Rb-MyoD pathway components including CBP and p300 [8], [9] and prolonged phosphorylation of Rb1 [8], which was associated with delayed MyoD activity and impaired skeletal muscle regeneration.

Skeletal muscle is composed of multi-nucleated, terminally differentiated myofibrils. Peripheral to these fibers is a niche populated by muscle stem cells called satellite cells or myogenic progenitors. Upon muscle damage quiescent satellite cells become activated. These activated satellite cells then asymmetrically divide to repopulate the niche and generate transient-amplifying myoblasts, which proliferate rapidly [13]. These myoblasts then differentiate into committed myocytes, which complete regeneration by fusing with the damaged myofibrils to repair the damaged skeletal muscle.

Several signaling pathways are important for muscle differentiation and regeneration, including the TGF-β [14], Wnt [15], [16], Notch [17], and IGF [18], [19], [20] pathways. An isoform of IGF-1 was shown to promote hypertrophy in adult mouse muscle through stimulating both muscle progenitor cell proliferation and enhanced differentiation of committed myoblasts [21]. During early myogenesis, high levels of IGF-1 increased expression of cell cycle promoting genes, including cyclins, and decreased expression of myotube specification genes, such as myogenin [22]. This was followed by decreased expression of cell cycle genes and increased expression of myogenesis promoting factors later in myogenesis [22]. Cells lacking a functioning IGF-1 receptor (IGF1R) show decreased expression of proliferation markers, including Ki67, and form fewer myotubes in culture [23], [24].

TGF-β-family signaling inhibits muscle development and regeneration, with inhibitory effects on both satellite cell recruitment and myoblast differentiation [25]. Addition of TGF-β1 in murine and human cell culture of C2C12 myoblasts blocked IGF1R signaling and inhibited IGF-II expression and excretion, resulting in the inhibition of muscle differentiation and muscle repair [26]. Decreased expression of important differentiation factors correlates with increased expression of myogenic progenitor proliferation factors in murine myoblasts treated with TGF-β [27].

Notch expression is upregulated in activated satellite cells and Notch signaling promotes satellite cell differentiation and increased numbers of proliferative myogenic progenitor cells. Addition of the constitutively active Notch intracellular domain was shown to repress initiation of the myogenic program by inactivating MyoD [28]. After differentiation into myogenic progenitor cells, high levels of Notch act to prevent further differentiation [17]. Further, actively dividing cells with high levels of Notch signaling were shown to retain their undifferentiated myogenic progenitor identity [17]. Decreased Notch signaling was also associated with muscle hypotrophy and loss of regeneration potential [29]. Thus Notch signaling is hypothesized to be integral for maintaining the satellite cell niche and myogenic progenitor cells.

Notch and Wnt signaling are temporally coordinated to ensure proper differentiation, as these pathways promote proliferation and differentiation, respectively. A switch from Notch to Wnt signaling is necessary during adult myogenesis [30]. Wnts and their downstream effector, β-catenin, promote progression from satellite cells to myotubes [31]. However, excessive Wnt signaling during aging ceases to be pro-myogenic and instead leads to increased muscular fibrosis [31], showing that inappropriate Wnt signaling during myogenesis is highly disruptive. In Wnt3a treated myoblasts, high levels of activated β-catenin are associated with differentiating but not proliferating cells [30]. Wnt3a has also been shown to promote myoblast fusion, an important step in myotube formation and muscle regeneration [32]. Frizzled7 and its related ligand Wnt7a were also shown to promote skeletal muscle regeneration by supporting the symmetric division of satellite cells, thereby increasing the pool of cells available for differentiation [33]. Interestingly, emerin was shown to interact with β-catenin and prevent its accumulation in the nucleus [34], suggesting that emerin regulates Wnt signaling in the myogenic lineage. Thus, we predict emerin-null myogenic progenitors will exhibit significantly perturbed Wnt signaling.

The importance of micro-RNAs (miRNAs), ∼22 nucleotide non-coding RNAs, as regulators of diverse cellular processes including development and differentiation has become evident in recent years [35]. miRNAs generally act by binding mRNAs at their 3′ UTR in the cytoplasm and degrading them or attenuating their rate of translation. Thus miRNAs are hypothesized to fine tune gene expression, which is crucial for regulating developmental pathways [36]. Several myogenic miRNAs (myomiRs) have been described as being particularly important in heart and skeletal muscle development, including miR-206 [37]. Myogenic regulatory factors Myf5 and Myogenin activate the expression of both miR-1 and miR-206, while MyoD is capable of activating only the expression of miR-206 [38]. miR-1 and miR-206 expression is upregulated early during myogenic differentiation and downregulated during regeneration in injured muscle [39]. miR-1 and miR-206 target Pax7 and regulate its expression [39]. Thus the coordinated expression of miR-1 and miR-206 is important for modulating the balance between proliferation and differentiation by directly regulating Pax7 [39]. miR-486 also regulates Pax7 expression and a miR-resistant form of Pax7 causes defective differentiation in myoblasts [40]. miR-221 and miR-222 are downregulated upon differentiation of proliferating myoblasts, then upregulated in terminally differentiated myotubes [41]. Ectopic expression of miR-221 and miR-222 are capable of disrupting early myogenesis and terminal myotube differentiation [41]. During myogenic differentiation, MyoD directly increases production of miR-378, which enhances MyoD activity by repressing the anti-myogenic protein MyoR [42]. miR-125b negatively regulates IGF-II expression, with excess levels acting to inhibit myoblast differentiation and muscle regeneration [43]. miR-181 is necessary for differentiation of myoblasts, with one of its targets being the myoblast differentiation repressor Hox-A11 [44]. miR-24 is upregulated during myoblast differentiation, and its expression is repressed by TGF-β1 in a Smad3-dependent manner [45].Though inroads have been made into the function of miRNAs in myogenesis, to date a complete picture of the miRNA-regulatory landscape in the myogenic lineage remains elusive. Further, changes in miRNA expression have only been determined in selected forms of muscular dystrophy models of Duchenne muscular dystrophy [46], [47], [48], [49]. It remains to be determined if miRNA expression changes in other forms of muscular dystrophy and how this may affect the transcriptome of skeletal muscle. Further, it will be important to determine how miRNA expression changes during satellite cell differentiation and muscle regeneration and how these expression changes are altered during differentiation and regeneration of dystrophic muscle.

To elucidate molecular pathways implicated in the EDMD disease mechanism, we performed mRNA and miRNA expression profiling in emerin-null myogenic progenitors. Emerin-null myogenic progenitors were used because the skeletal muscle phenotype of EDMD is predicted to be caused by the failure of myogenic progenitors to differentiate properly and regenerate damaged muscles [8], [9], [50]. Although gene expression profiling has been performed in muscle from patients and mice, these tissues are a heterogeneous population of cells containing fully differentiated cells, muscle stem cells, myogenic progenitors, fibroblasts and immune infiltrate. Thus the use of cultured myogenic progenitors has significant advantages over whole tissue analysis. We found that several components of the Notch, Wnt, TGF-β, and IGF pathways were misexpressed in emerin-null progenitors. Downstream targets of these pathways were also misexpressed at both the transcriptional and translational level. Phosphorylation of p38, a key component of these signaling pathways, was also altered in emerin-null myogenic progenitors. Perturbations in all four pathways suggest that the loss of emerin has significant effects on myogenic signaling in murine myogenic progenitors.

## Results

### Gene Expression Profiling of Emerin-null Myogenic Progenitors

We performed whole genome mRNA microarray analysis on proliferating wildtype and emerin-null H2K myogenic progenitors to determine how myogenic gene expression was affected by emerin loss. H2K myogenic progenitors were used for this analysis because myogenic progenitors derived from emerin-null H2K mice are superior to the C2C12 myoblast-derived cell line in many ways. For example, decades in cell culture have caused C2C12 myoblasts to diverge significantly from the myoblasts they were derived from and are aneuploid for multiple chromosomes. Emerin-null H2K myogenic progenitors conditionally express the SV40 large-T antigen, which allows primary cells derived from these mice to be passaged directly for multiple generations without losing their progenitor identity [51], [52]. H2K myogenic progenitors can be efficiently induced to differentiate into myotubes upon serum withdrawal.

**Figure 1:**
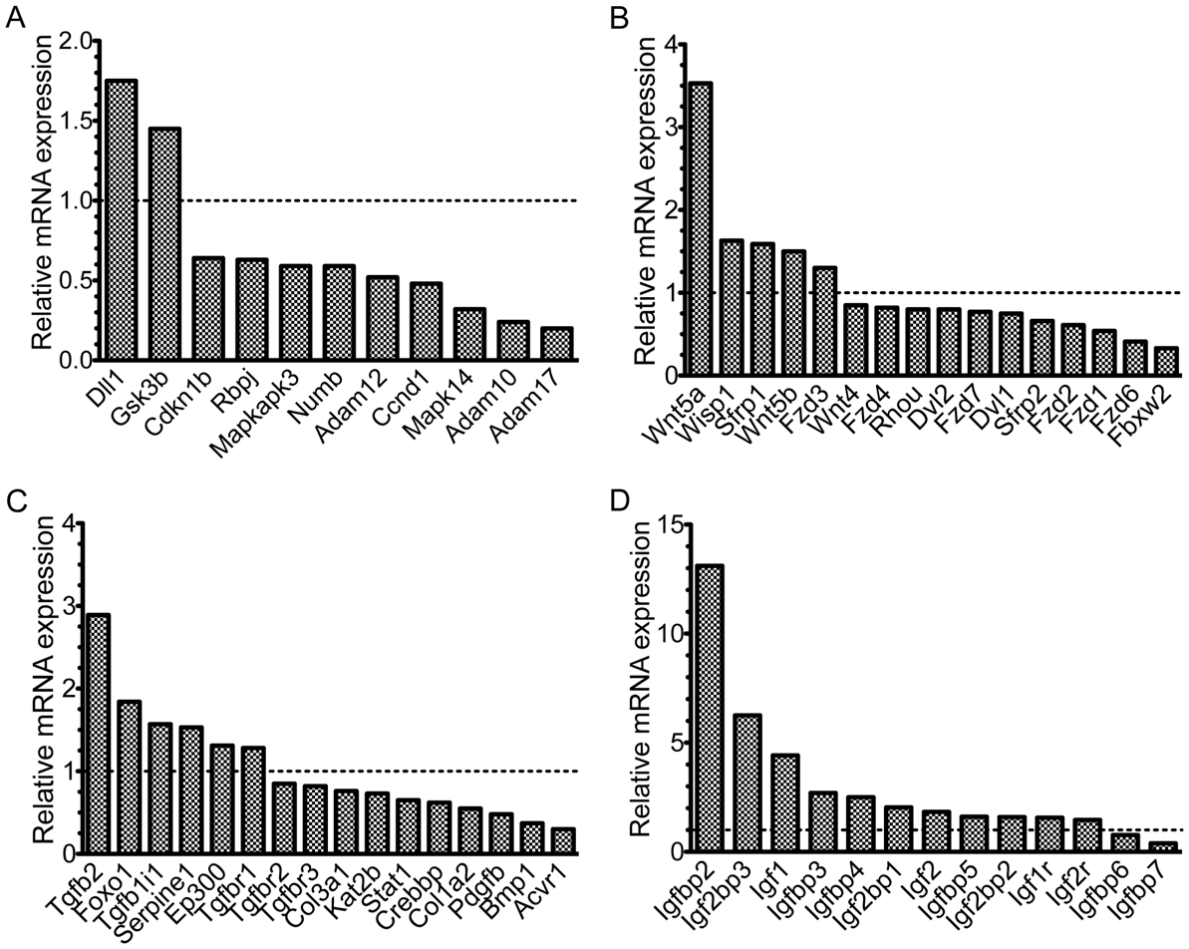
mRNA expression profiling identifies changes in Notch, Wnt, TGF-β and IGF pathways. mRNA microarray expression data from emerin-null myogenic progenitors was normalized to mRNA expression in wildtype progenitors. Dotted line represents no change in expression. A) Notch pathway. B) Wnt pathway. C) TGF-β pathway. D) IGF pathway. All changes in gene expression were statistically significant (p≤0.05).

RNA was isolated from 500,000 wildtype or emerin-null myogenic progenitors and hybridized to Affymetrix Mouse Whole Genome 430 2.0 microarrays (n = 2). The microarray data is available through the NCBI Gene Expression Omnibus and accessible through GEO accession numbers GSM787509–GSM787512. This analysis showed extensive differences in gene expression between wildtype and emerin-null myogenic progenitors, as 13.3% of genes (p<0.05, fold-change >1.5) showed perturbed expression in emerin-null H2K myogenic progenitors. QVALUE software developed by Alan Dabney and John Storey was then used to increase the confidence in these results. Using this approach we found that a p-value <0.035 was required to obtain a False Discovery Rate (FDR) of 5%. Applying this threshold to our data results in 2792 genes out of 21804 unique transcripts (12.5%) altering mRNA expression by greater than 1.5-fold in emerin-null H2K myogenic progenitors. mRNA with altered expression in emerin-null myogenic progenitors included components of the CREBBP/p300 transcriptional pathway, including CREBBP, Ep300 and Kat2b (Figure 1C). Studies previously found perturbed expression of components of the CREBBP/p300 transcriptional pathway in emerin-null human [9] and mouse [8] skeletal muscle. Emerin-null skeletal muscle and emerin ablation in myoblasts also altered the expression of MyoD [8], [50], an essential myogenesis promoting factor. We also recently showed altered MyoD expression in emerin-null myogenic progenitors [53].

### Gene Networks Affected by Loss of Emerin Include Components of Signaling Pathways that Regulate Myogenic Progenitor Proliferation and Differentiation

Microarray expression data was analyzed with Ingenuity software to identify gene networks and pathways significantly affected by loss of emerin. This analysis showed there was significant perturbation of signaling pathways previously shown to be important for regulating myogenic differentiation, including Notch, Wnt, TGF-β, and IGF (Figure 1; Figure S1, S2, S3, S4). We found the Notch pathway components *Dll1* (1.74-fold) and *Gsk3b* (1.45-fold) were upregulated in emerin-null myogenic progenitors, while *Ckn1b* (0.64-fold), *Rbpj* (0.63-fold), *Mapkapk3* (0.59-fold), *Numb* (0.59-fold), *Adam12* (0.52-fold), *Ccnd1* (0.48-fold), *Mapk14*/p38 (0.32-fold), *Adam10* (0.24-fold), and *Adam17* (0.20-fold) were downregulated (Figure 1A). Downregulation of these genes suggests Notch signaling is attenuated in emerin-null progenitors. However, the Notch antagonist *numb* is also downregulated, suggesting compensatory changes in expression of Notch pathway inhibitors may result in normal Notch signaling in the absence of emerin.

Expression of Wnt pathway components was also altered in emerin-null myogenic progenitors (Figure 1B). Wnt pathway genes *Wnt5a* (3.53-fold), *Wisp1* (1.63-fold), *Sfrp1* (1.59-fold), *Wnt5b* (1.5-fold), and *Fzd3* (1.3-fold) were all upregulated. Wnt pathway transcripts *Wnt4* (0.85-fold), *Fzd4* (0.82-fold), *Rhou* (0.80-fold), *Dvl2* (0.80-fold), *Fzd7* (0.77-fold), *Dvl1* (0.75-fold), *Sfrp2* (0.66-fold), *Fzd2* (0.61-fold), *Fzd1* (0.54-fold), *Fzd6* (0.41-fold), and *Fbxw2* (0.33-fold) were downregulated. Decreased expression of several Wnt receptors suggests that Wnt signal transduction may be impaired in emerin-null myogenic progenitors.

Expression of TGF-β pathway components was altered in emerin-null myogenic progenitors (Figure 1C). *Tgfb2* (2.89-fold), *Foxo1* (1.84-fold), *Tgfb1i1* (1.57-fold), *Serpine1* (1.53-fold), *Ep300*/p300 (1.31-fold) and *Tgfbr1* (1.28-fold) were upregulated. *Tgfbr2* (0.85-fold), *Tgfbr3* (0.82-fold), *Col3a1* (0.76-fold), *Kat2b*/PCAF (0.73-fold), *Stat1* (0.65-fold), *Crebbp*/CBP/p300 (0.62-fold), *Col1a2* (0.55-fold), *Pdgfb* (0.48-fold), *Bmp1* (0.37-fold), and *Acvr1* (0.3-fold) were downregulated. These results suggest that TGF-βsignaling is attenuated in emerin-null myogenic progenitors, resulting in impaired activation of downstream target genes.

Emerin-null myogenic progenitors exhibit altered expression of IGF pathway genes (Figure 1D). *Igfbp2* (13.11-fold), *Igf2bp3* (6.25-fold), *Igf1* (4.42-fold), *Igfbp3* (2.7-fold), *Igfbp4* (2.5-fold), *Igf2bp1* (2.04-fold), *Igf2* (1.83-fold), *Igfbp5* (1.61-fold), *Igf2bp2* (1.59-fold), *Igf1r* (1.57-fold) and *Igf2r* (1.46-fold) were upregulated. *Igfbp6* (0.77-fold) and *Igfbp7* (0.39-fold) were downregulated. While *Igf1* and *Igf2* are upregulated, numerous IGF-binding proteins appear to be concomitantly upregulated, suggesting emerin-null progenitors are attempting to compensate for increased IGF signaling. From this expression data it cannot be predicted whether IGF signaling is disrupted in emerin-null myogenic progenitors.

### Validation of Selected Pathway Components

**Figure 2:**
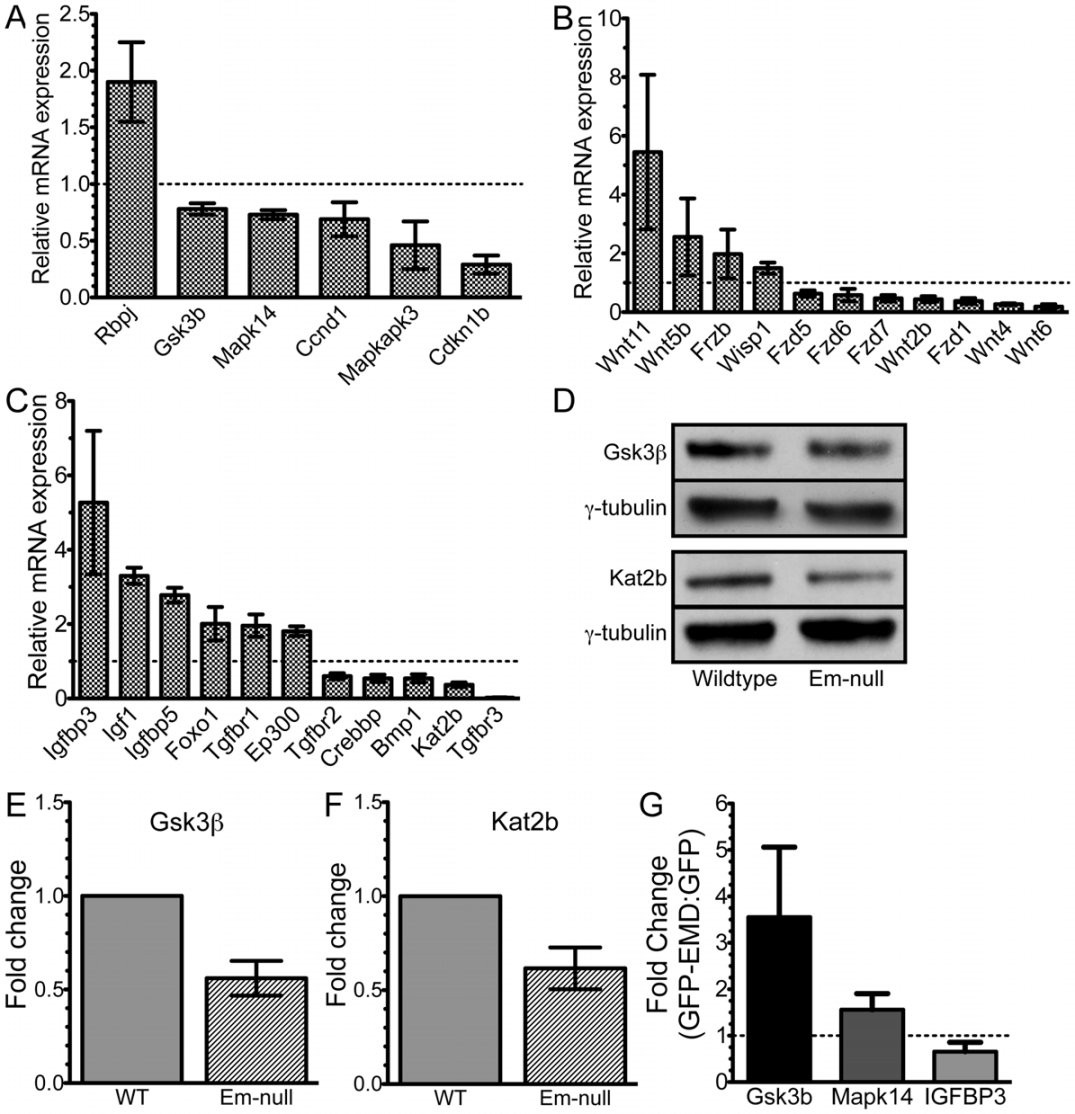
Validation of selected components of the Notch, Wnt, TGF-β and IGF pathways. Validation of mRNA expression changes of selected Notch, Wnt, TGF-β and IGF pathways by qPCR. Dotted line represents no change in expression. A) Notch and p38 components. B) Wnt components. C) IGF and TGF-β components. All changes in gene expression were statistically significant (p≤0.05). D) Whole cell lysates of wildtype and emerin-null H2K myogenic progenitors were separated by SDS-PAGE and western-blotted for GSK3β, Kat2b and γ-tubulin. E,F) Densitometry was performed on blots in panel D and GSK3β and Kat2b levels were normalized to γ-tubulin. Emerin-null protein expression was normalized to protein expression in wildtype progenitors. G) GFP-emerin or GFP was expressed in emerin-null cells and *Gsk3b*, *Mapk14*, *Igfbp3* and *Gapdh* were measured by qPCR. Data is depicted as fold-change in GFP-emerin expressing cells compared to GFP-transfected cells.

Quantitative RT-PCR (qPCR) was used to validate 49 components of the Notch, Wnt, TGF-β, and IGF signaling pathways. Supporting the microarray results, we found the Notch pathway component *Rbpj* (1.9-fold) was upregulated and *Notch*, *Gsk3b* (0.78-fold), *Mapk14*/p38 (0.73-fold), *Ccnd1* (0.69-fold), *Mapkapk3* (0.46-fold) and *Cdkn1b* (0.29-fold) were downregulated (Figure 2A). Wnt pathway components *Wnt11* (5.45-fold), *Wnt5b* (2.56-fold), *Frzb* (1.98-fold), and *Wisp1* (1.5-fold) were upregulated in emerin-null myogenic progenitors (Figure 2B). Emerin-null progenitors had decreased expression of *Fzd1* (0.38-fold), *Fzd5* (0.63-fold), *Fzd6* (0.58-fold), *Fzd7* (0.47-fold), *Wnt2b* (0.44-fold), *Wnt4* (0.27-fold) and *Wnt6* (0.19-fold). Components of the TGF-β and IGF pathways were also validated by qPCR, as *Igfbp3* (5.27-fold), *Igfbp5* (2.78-fold), *Igf1* (3.3-fold), *Tgfbr1* (1.96-fold), *Foxo1* (2.01-fold) and *Ep300*/p300 (1.81-fold-fold) were upregulated and *Tgfbr2* (0.6-fold), *Crebbp*/CBP/p300 (0.54-fold), *Bmp1* (0.54-fold), *Kat2b*/PCAF (0.37-fold) and *Tgfbr3* (0.02-fold) were downregulated (Figure 2C).

GFP-emerin or GFP was expressed in emerin-null myogenic progenitors and *Gsk3b*, *Mapk14* and *Igfbp3* expression was measured to test if emerin was directly responsible for their altered gene expression. Emerin was overexpressed 601-fold, which is similar to the amount of decreased emerin expression seen in emerin-null myogenic progenitors (839-fold). mRNA levels were normalized to *Gapdh* expression. Changes in *Gsk3b*, *Mapk14* and *Igfbp3* mRNA expression were determined by comparing their expression in GFP-emerin expressing cells to GFP transfected cells. Importantly, expression of GFP-emerin increased the expression of *Gsk3b* (3.55±1.5 fold) and *Mapk14* (1.56±0.35 fold) and decreased *Igfbp3* expression (0.65±0.2 fold) in emerin-null cells (Figure 2G). Thus loss of emerin is directly responsible for altered expression of *Gsk3b*, *Mapk14* and *Igfbp3* in emerin-null cells.

Kat2b and GSK3β protein levels were then measured to confirm changes in mRNA expression resulted in decreased protein expression. Cell lysates from wildtype and emerin-null myogenic progenitors were separated by SDS-PAGE, transferred to nitrocellulose and western blotted for Kat2b, GSK3β and γ-tubulin. γ-tubulin was the loading control. Densitometry was used to determine Kat2b and GSK3β protein expression. Importantly, GSK3β and Kat2b protein expression was reduced 0.56±0.12 fold (n = 5; Figure 2D,E) and 0.66±0.07 fold (n = 4; Figure 2D,F), respectively. Thus, reduced Kat2b and GSK3β mRNA expression results in reduced Kat2b and GSK3β protein expression.

It was formally possible that conditional expression of the Large T-antigen in the H2K myoblasts was responsible for the altered expression of these signaling components. Primary myoblasts were derived from wildtype C57BL/6 and protein levels of Myf5, GSK3β and Kat2b were measured to test if their expression was altered in H2K myoblasts. Whole cell lysates of primary myoblasts and wildtype H2K myoblasts were separated by SDS-PAGE and western blotted for Myf5, Kat2b, GSK3β and γ-tubulin. Importantly, there was no difference in expression of Myf5, GSK3β and Kat2b between primary myoblasts and wildtype H2K myoblasts (Figure S5).

**Figure 3:**
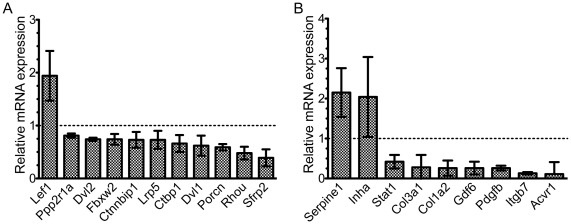
Downstream target genes of the Wnt and TGF-β are also altered in emerin-null myogenic progenitors. mRNA expression of selected downstream targets of the Wnt and TGF-β pathways was measured using qPCR. Dotted line represents no change in expression. A) Wnt targets. B) TGF-β targets. All changes in gene expression were statistically significant (p≤0.05).

To determine whether changes in expression of Notch, IGF-1, TGF-β or Wnt pathway components affected downstream signaling, we measured the expression of selected target genes within these pathways. Importantly, we found significant changes in the expression of downstream Wnt targets, including *Lef1* (1.94-fold), *Ppp2r1a* (0.81-fold), *Dlv2* (0.74-fold), *Fbxw2* (0.74-fold), *Ctnnbip1* (0.73-fold), *Lrp5* (0.73-fold), *Ctbp1* (0.66-fold), *Dvl1* (0.62-fold), *Porcn* (0.59-fold), *Rhou* (0.48-fold), and *Sfrp2* (0.39-fold; Figure 3A). Decreased activation of these Wnt target genes is consistent with attenuation of Wnt signaling in emerin-null myogenic progenitors.

**Figure 4:**
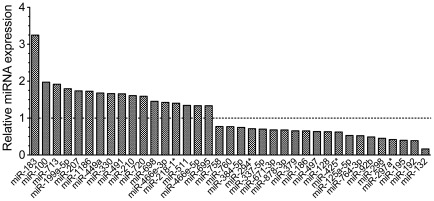
Expression of miRNAs was altered in emerin-null myogenic progenitors. miRNA expression was determined for emerin-null and wildtype myogenic progenitors by microarray. miRNA expression in emerin-null myogenic progenitors was normalized to wildtype miRNA expression in wildtype progenitors. Dotted line represents no change in expression. All changes in expression were statistically significant (p≤0.05).

Changes in expression of TGF-β pathway components in emerin-null myogenic progenitors also altered the expression of target genes, including *Serpine1* (2.15-fold), *Inha* (2.04-fold), *Stat1* (0.42-fold), *Col3a1* (0.28-fold), *Col1a2* (0.26-fold), *Gdf6* (0.26-fold), *Pdgfb* (0.26-fold), *Itgb7* (0.13-fold), and *Acvr1* (0.11-fold; Figure 4b). These results show that emerin-null progenitors have attenuated TGF-β signaling, as a majority of these target genes were expressed at lower levels than in wildtype progenitors. Increased expression of certain TGF-β signaling components or target genes may reflect a mechanism for these cells to attempt to compensate for decreased TGF-β signaling.

### Emerin-null Myogenic Progenitors Show Disrupted miRNA Expression

We next examined miRNA expression in wildtype and emerin-null myogenic progenitors, as miRNA expression profiling had never been examined in emerin-null skeletal muscle or isolated myogenic progenitors. miRNAs are often contained within RNA polymerase II transcripts and thus we predicted emerin-null progenitors would exhibit changes in miRNA expression. To determine if emerin-null myogenic progenitors had disrupted miRNA expression, we performed miRNA expression analysis using Affymetrix Genechip miRNA microarrays. The microarray data is accessible through GEO accession numbers GSM787505-GSM787508. Though the Genechip miRNA array contains probes for all known miRNAs across multiple species, we chose to limit our analysis to the 609 mouse miRNAs, as sequence homology between miRNAs of different species is an imperfect predictor of shared targets. Of the mouse miRNAs on the chip, 47 showed altered expression levels in emerin-null progenitors (p<0.05), representing 7.7% of all known murine miRNAs. We used the MicroCosm Targets algorithm to identify potential mRNA targets. Interestingly, many of these miRNAs are predicted to target myogenic signaling components, some of which also showed aberrant mRNA expression (Table 1).

**Table 1:** Selected miRs and their predicted targets, as determined by MicroCosm Targets.

miRNA	Predicted Targets
miR-100	Fgf20, Fzd8, Hdac3, Myot, Smad7, Bmp3, Rbbp5
miR-132	Hdac3, Smad2, Wnt2, Gsk3a, Fgf21, Nup98, Mapk3, Wnt10a, Dll4
miR-183	Hdac11, Foxp1, Map2k1
miR-192	Ctcf, Myot, Wnt7a, Igf2, Rbpj, Mapk10, Serpine2
miR-195	Smad1, Fgf8, Wnt8a, Myo5b, Smad7, Smad2, Epha1, Mapk3, Fzd3
miR-199a-5p	Map3k11, Foxp1, Unc45a, Fgf4, Igfbp2, Foxo4, Hdac2, Sirt1
miR-207	Map3k11, Lmnb1, Numbl, Map3k10, Ncor2, Igfbp2, Igfbp5, Inha, Map3k5, Igf1
miR-297a*	Lmnb1, Nup98, Fgfr3, Epha5, Mapkapk2
miR-298	Igf1r, Igf1, Myf5, Mapk15, Itgb5, Map2k5, Adam15, Mapk13, Cdkn1a, Map3k3
miR-713	Epha1, Tgfa, Mapk14, Akt1

Of particular note are some of the most highly dysregulated miRNAs, including miR-183 (3.25-fold), miR-100 (1.97-fold), miR-713 (1.92-fold), miR-199a-5p (1.8-fold), miR-207 (1.74-fold), miR-132 (0.17-fold), miR-192 (0.39-fold), miR-195 (0.40-fold), miR-297a* (0.42-fold) and miR-298 (0.45-fold; Figure 4). Importantly, these are predicted to target important signaling components in the Notch, Wnt, TGF-β, and IGF pathways (Table 1). While several of the hits from our mRNA microarray studies appear to also be targets of post-transcriptional regulation, including Igf1, Igf2, Inha, Igfbp5, Fzd3, Wnt10a, Rbpj and Mapk14, we also report here several interesting putative miRNA targets that may have disrupted protein expression in the absence of emerin, including Lamin B1, the insulator protein CTCF, and HDAC3.

**Figure 5:**
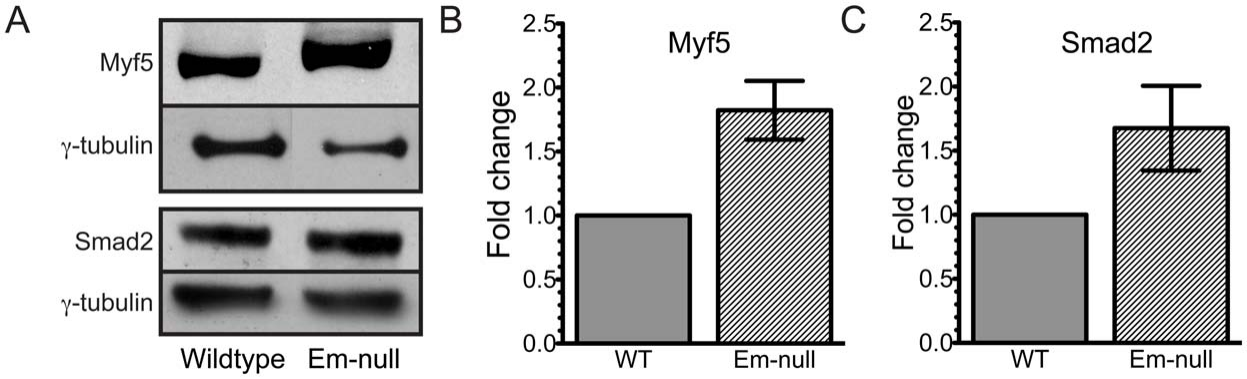
miRNA-downregulation causes increased expression of target proteins. A) Whole cell lysate was separated by SDS-PAGE and western-blotted for Myf5, Smad2 and γ-tubulin. B,C) Densitometry was done on blots in panel A and Myf5 and Smad2 were normalized to γ-tubulin. Protein expression in wildtype myogenic progenitors was set to one.

Decreased expression of miR-298 and miR-195 is predicted to result in increased protein expression of Myf5 and Smad2, respectively, in emerin-null myogenic progenitors. Our previous work showed that MyoD and Myf5 were upregulated in emerin-null myogenic progenitors [53]. Myf5 and MyoD expression was rescued by exogenous expression of emerin in these emerin-null progenitors. Western blotting was performed on wildtype and emerin-null myogenic progenitors to confirm Myf5 and Smad2 expression was increased in emerin-null myogenic progenitors. Myf5 and Smad2 protein levels were normalized to γ-tubulin levels. Confirming our previous results, Myf5 protein levels increased 1.82±0.22 fold (n = 5) in emerin-null myogenic progenitors (Figure 5A,B). Smad2 protein levels increased 1.68±0.3 (n = 4; Figure 5A,C). Thus, reduction of miR-298 and miR-195 results in increased expression of their predicted targets.

**Figure 6:**
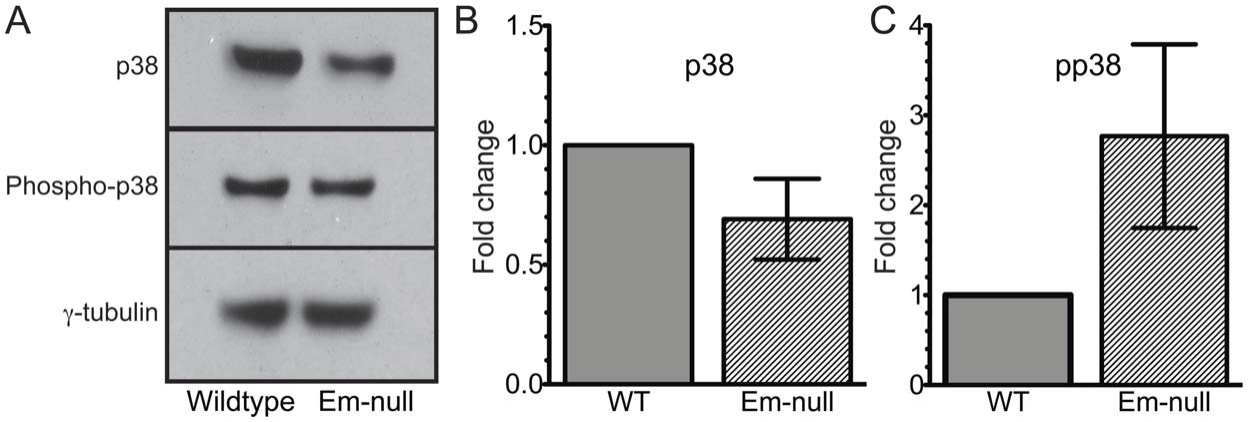
IGF signaling is activated in emerin-null myogenic progenitors. Protein expression of p38 and phosphorylated p38 in emerin-null myogenic progenitors was monitored by western blotting (A) and quantitated by densitometry normalized to γ-tubulin (B) or total p38 (C) levels in wildtype progenitors. Antibodies specific for p38 (B) or phosphorylated p38 (C) were used to monitor activation of the IGF-1 pathway.

Upregulation of *miR-713* is likely to lead to decreased p38 protein and a diminished ability of emerin-null myogenic progenitors to respond to external p38 activation stimuli, since *miR-713* is predicted to target Mapk14/p38. This is significant because recent evidence shows p38 is directly responsible for repression of Pax7 after satellite cell expansion during muscular regeneration [54], [55]. To test if Mapk14/p38 protein levels are decreased in emerin-null myogenic progenitors we performed western blotting on wildtype and emerin-null progenitor cell lysates using antibodies against Mapk14/p38 (Figure 6A). Emerin-null myogenic progenitors exhibit 0.65-fold expression of Mapk14/p38 protein compared to wildtype (Figure 6B). Interestingly, our mRNA microarray analysis showed Pax7 expression was also increased 2.9-fold in emerin-null progenitors, suggesting that the downregulation of p38 is physiologically relevant in emerin-null myogenic progenitors.

### Disrupted Protein Expression and Signaling in Emerin-null Myogenic Progenitors

To test if IGF signaling was activated in emerin-null myogenic progenitors, as predicted from expression profiling, proliferating progenitors were incubated with 10 ng/ml recombinant IGF-1 for 10 minutes before harvesting. Cell lysates were separated by SDS-PAGE and western blotted using antibodies specific for p38 or phospho-p38. As expected, emerin-null myogenic progenitors increased phospho-p38 levels 3.1±0.9-fold (Figure 6C). When phospho-p38 levels are normalized to total p38, the emerin-null myogenic progenitors increase total phospho-p38 two-fold in response to IGF. Thus increased IGF binding protein expression fails to completely compensate for increased expression of IGF-I, IGF-II and the IGF receptors.

**Figure 7:**
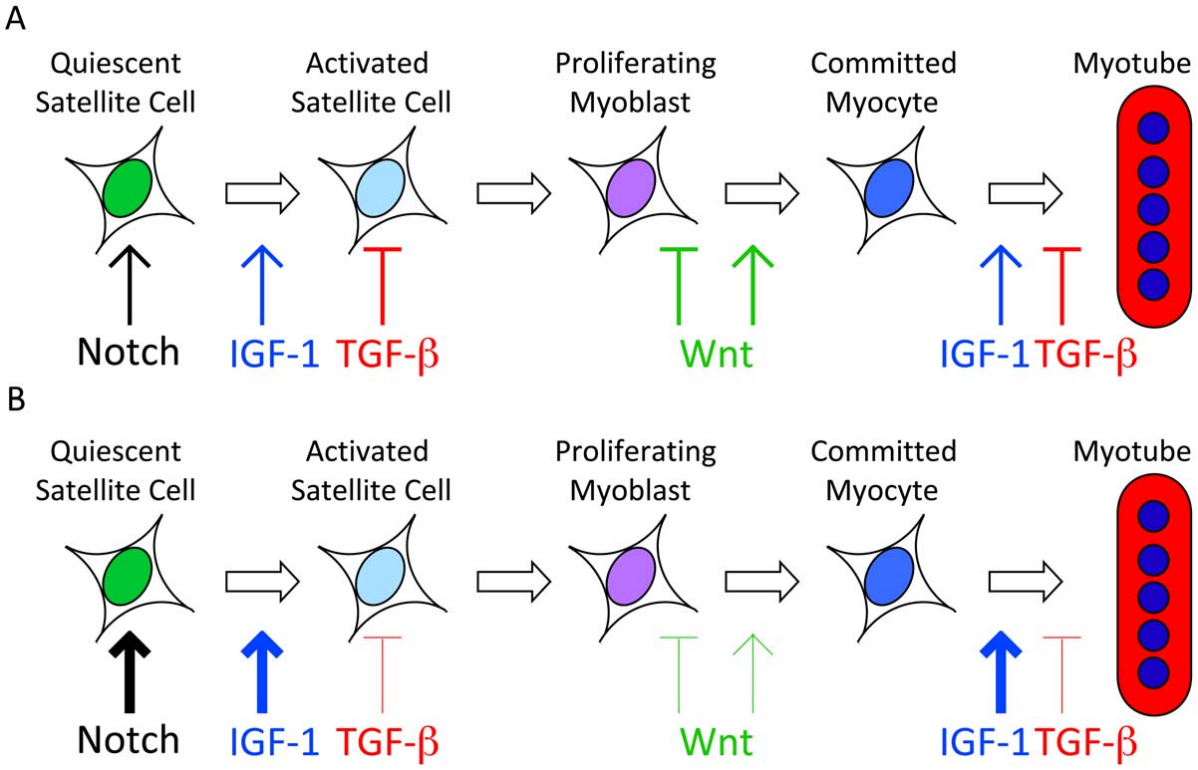
Model of predicted effects of emerin loss on myogenic signaling during differentiation. A) Notch, Wnt, TGF-β and IGF signaling act at specific times during myogenic differentiation to ensure proper differentiation. B) Our data predicts that activation of Notch and IGF-1 signaling pathways and attenuation of Wnt and TGF-β signaling pathways will lead to impaired differentiation by maintaining the progenitors in a proliferative state and exhausting the satellite cell niche.

Collectively, these findings indicate that emerin-null myogenic progenitors exhibit altered Wnt, TGF-β, Notch and IGF-1 signaling and predicts that emerin-null myogenic progenitors exhibit enhanced proliferation and decreased differentiation (Figure 7). Increased myoblast proliferation and decreased differentiation and muscle regeneration was previously reported for primary myoblasts derived from emerin-null mice [8]. This study significantly advances our understanding of the molecular mechanism underlying decreased muscle regeneration seen in emerin-null mice.

## Discussion

We report here major disruptions in the Notch, Wnt, TGF-β and IGF pathways in myogenic progenitors derived from emerin-null mice. Disruption of these pathways is predicted to cause impaired myogenic differentiation (Figure 7). Previous studies showed defective Rb signaling in skeletal muscle from EDMD patients and emerin-null mice, which resulted in increased MyoD expression and delayed myogenic differentiation and gene expression [8], [9]. However, this is the first report using genome-wide expression profiling of mRNA and miRNA to identify and begin interrogating broad signaling defects in emerin-null myogenic progenitors. Our data represent compelling evidence that loss of a single nuclear envelope protein can have drastic consequences on the expression of hundreds of genes, many of which play important roles in myogenic differentiation and muscle regeneration. With an ever-growing number of nuclear envelope transmembrane proteins (NETs) being identified, it will be essential to characterize their individual functions in detail and determine their relative contributions to transcriptional and signaling pathways.

### Myogenic Signaling and the EDMD Mechanism

Notch signaling maintains the undifferentiated state of satellite cells [30] and a switch from Notch to Wnt signaling is necessary for myogenic progenitors to begin differentiating and repair damaged muscle tissue [30]. The increase in Notch signaling in emerin-null progenitors may interfere with myogenesis by blocking this transition and preventing the activation of quiescent satellite cells in vivo. We predict the inability to activate sufficient numbers of satellite cells in emerin-null skeletal muscle may result in decreased numbers of proliferative myogenic progenitors and myoblasts and contribute to the decreased regenerative potential seen in EDMD.

Excessive canonical Wnt signaling negatively affects the regenerative capacity of cultured satellite cells by converting them to a fibrogenic lineage [31]. High levels of Wnt signaling were also shown to increase fibrosis of aged skeletal muscle [31]. Wnt signaling acts downstream of Notch signaling to promote differentiation during adult myogenesis [30], and the Wnt effector β-catenin promotes satellite cell to myotube progression [31]. With a perturbation of the temporal switch from Notch to Wnt signaling [30], we predict there is an increase in the pool of proliferating myoblasts during differentiation of emerin-null myogenic progenitors. Based upon the decrease in Wnt signaling, we also predict an impaired ability of emerin-null myogenic progenitors to differentiate into committed myocytes and myotubes. We hypothesize that increased myoblast proliferation and failure to differentiate contributes to the impaired regeneration seen in EDMD.

TGF-β acts early in myogenesis to prevent activation of promyogenic genes in satellite cells and to antagonize terminal differentiation and fusion of myocytes to myotubes [14], [56]. Aberrantly high TGF-β signaling was shown to interfere with muscle regeneration by increasing phosphorylation of SMAD3 in satellite cells and interfering with Notch activity [57]. TGF-β signaling was downregulated in emerin-null myogenic progenitors. Thus we predict that activation of satellite cells and myocyte cell fusion to the damaged muscle fiber may be enhanced in skeletal muscle lacking emerin. These effects are predicted to partially mitigate the increased proliferation and block of differentiation caused by increased Notch and Wnt signaling in emerin-null myogenic progenitors. However, the degree to which downregulation of TGF-β may mitigate the increased proliferation and differentiation block of emerin-null progenitors remains to be determined.

IGF signaling is mitogenic, as it promotes satellite cell proliferation early in differentiation and promotes terminal differentiation of myocytes during skeletal muscle regeneration [58]. IGF signaling was enhanced in proliferative emerin-null myoblasts and we predict that in emerin-null skeletal muscle quiescent satellite cells are pushed towards an activated state, thereby depleting the quiescent satellite cell niche. Further, decreased TGF-β signaling is predicted to synergize with increased IGF-1 signaling to deplete the pool of quiescent satellite cells. We further predict increased fusion of myocytes to myotubes would also been seen in emerin-null skeletal muscle. However, it will be necessary to determine if perturbed IGF signaling persists at similar levels during the entirety of myogenesis in emerin-null cells to influence myocyte fusion.

p38 signaling was shown to inhibit the expression of Pax7 by regulating the formation of the polycomb repressive complex 2 (PRC2) at the Pax7 promoter [54], [55]. p38 phosphorylates the EZH2 subunit of PRC2, which stabilizes the interaction between PRC2 and the Pax7 promoter, resulting in promoter repression. Pax7 confers myogenic lineage identity and is necessary for maintenance of the satellite cell niche [59]. Pax7 also mediates the initial activation of myogenic progenitor proliferation and thus is an essential regulator of myogenic differentiation [55]. Emerin-null myogenic progenitors have increased phospho-p38, suggesting activation of phospho-p38 signaling, which is predicted to suppress Pax7 expression and impair differentiation. This coupled to decreased pro-myogenic Wnt signaling in emerin-null myogenic progenitors suggests emerin-null myogenic progenitors have increased proliferative potential and impaired differentiation into committed myocytes. Loss of p38 also inhibits IGF-II expression and impairs myoblast differentiation [60], which we predict contributes to impaired regeneration in EDMD.

Collectively, perturbation of these signaling pathways may help explain the relatively late onset (2^nd^ or 3^rd^ decade of life; [61]) of skeletal muscle wasting seen in EDMD, since progressive loss of the satellite cell niche is predicted to cause a relatively slow loss of muscle regeneration over time. Additionally the pathogenesis of the disease may occur slowly because increased or decreased signaling in other partially redundant pathways may compensate for defects in the signaling pathways above. This may also explain why the dystrophic phenotype of EDMD tends to have a later onset than other forms of muscular dystrophy.

### Disrupted miRNA Expression

In several instances, such as miR-713, perturbed miRNA expression appears to be related to the disrupted expression of myogenic signaling components (e.g., p38). Several miRs that were shown to play roles in muscle differentiation, including miR-1 and miR-206, did not show disrupted expression in emerin-null myogenic progenitors. While myomiR expression is driven by myogenic regulatory factor expression, our studies do not show a direct relationship between perturbed myogenic regulatory signaling and disrupted expression of myomiRs in emerin-null myogenic precursors. Though it is now possible to acquire genome-wide miRNA expression data, the complexity of interactions between many miRs that target a single transcript, as well as the hundreds of transcripts that are targeted by a single miR make the annotation and validation of miR targeting an important goal for the future. Future studies will also analyze how changes in expression of miRs in emerin-null myogenic progenitors affect cell signaling and transcriptional pathways important for myogenic differentiation and skeletal muscle regeneration.

Now that we have identified perturbations of the Notch, Wnt, TGF-β and IGF pathways in emerin-null myogenic progenitors, it will be important to test if impaired signaling persists during differentiation of emerin-null progenitors. It will also be important to determine the relative contributions of each signaling pathway to the impaired differentiation and muscle regeneration seen in emerin-null myogenic progenitors. Further studies also need to examine whether using pharmacological inhibitors or activators of these important myogenic signaling pathways are able to restore normal signaling pathway function in emerin-null myogenic progenitors. These compounds would then be candidates for treating the impaired muscle regeneration in emerin-null mice and EDMD patients. Using this approach we predict potential drugs will be identified to treat EDMD patients.

## Materials and Methods

### Myogenic Progenitor Isolation and Cell Culture

Wildtype and Emerin-null H2K mouse myogenic progenitors were a generous gift from Tatiana Cohen and Terry Partridge (Children’s National Medical Center, Washington, DC). Emerin-null H2K mice were generated by Tatiana Cohen and Terry Partridge by breeding emerin-null and H-2K^b^tsA58 mice [51], [52]. Myogenic progenitors were isolated and maintained as previously described [52], [53]. Briefly, extensor digitorum longus (EDL) muscles were isolated and placed into 2 mg/ml collagenase (Sigma, product #C0130) in DMEM (Invitrogen, product #11995-065) for 1–2 hours at 35°C. Individual fibers were then isolated and each fiber was transferred serially through 2–4 petri dishes containing DMEM to select for undamaged fibers. Fibers were placed into matrigel-coated petri dishes containing DMEM, 10% horse serum (Invitrogen product #16050-098), 0.5% chick embryo extract (Accurate Chemical, product #CE6507), 2% L-Glutamine (Invitrogen, product #25030-081) and 1% penicillin/streptomycin (Invitrogen, product #15140-122) for 3–4 hours at 37°C. Myogenic progenitors were isolated from individual fibers by transferring each fiber into one matrigel-coated well of a 24-well plate containing proliferation media consisting of DMEM, 20% Heat inactivated FBS (Invitrogen, product #10082-147), 2% chick embryo extract, 2% L-glutamine, 1% pen-strep and 20 ng/ml γ-Interferon (Millipore, product #IF005). The fibers were incubated for 24–48 hours at 33°C, 10% CO_2_. Upon attachment of a single myogenic progenitor to the well, the fiber was removed and the myogenic progenitor was incubated in proliferation media for another 48 hours at 33°C, 10% CO_2_. Approximately 200 cells are expected after 48 hours and these were split and proliferated until enough cells were obtained for our analyses. H2K myogenic progenitors were maintained in proliferation media at 33°C and 10% CO_2_. Cells between passages 4–10 were used for these studies.

### Genome-wide Expression

Total RNA was isolated from 500,000 sub-confluent proliferative wildtype or emerin-null H2Ks using the miRNeasy Mini Kit (Qiagen, product #217004) and processed according to manufacturer’s protocol. RNA was isolated from two independent cell culture plates for each replicate to determine significant changes in mRNA and miRNA expression. Each isolated RNA sample was then hybridized to GeneChip Mouse Genome 430 2.0 Arrays (Affymetrix) at the University of Chicago Functional Genomics Facility. The Affymetrix statistical software suite was used for statistical analysis using Robust Multichip Analysis at the Functional Genomics Facility to identify significant differences between both samples. To identify changes in miRNA expression 1 ug of total RNA was hybridized to GeneChip miRNA Arrays (Affymetrix) and processed at the University of Chicago Functional Genomics Facility. Principal component analysis was performed on the samples and the two conditions segregated into two independent and defined groups. Statistical analysis using Robust Multichip Analysis was performed by the Functional Genomics Facility to identify significant differences between both samples. The microarray data is MIAME compliant and is available through the NCBI Gene Expression Omnibus, accessible through GEO accession numbers GSM787505-GSM787512. Selected mRNA levels were confirmed by qPCR, as described below.

### qPCR

Total RNA was prepared as above and cDNA was generated from RNA using MMLV Reverse Transcriptase (Invitrogen, product # 28025013) per manufacturer instructions for individual qPCRs. For qPCR arrays, cDNA was generated using the RT^2^ First Strand Kit (SABiosciences, product #C-03). qPCR was performed using a combination of individual primer sets (see Table 2 for sequences of primers used and SYBR GreenER qPCR SuperMix (Invitrogen, product #11761-500). RT^2^ Profiler PCR arrays were also used for the Mouse Wnt (SABiosciences, product #PAMM-043A-2) and TGF-β/BMP (SABiosciences, product #PAMM-035A-2) pathways using RT^2^ Real-Time SYBR Green/fluorescein PCR master mix (SABiosciences, product #PA-011).

**Table 2:** List of qRT-PCR primers used in this study.

Gene	Forward (5′-3′)	Reverse (5′-3′)
Bmp1	GCCATCCATCAAAGCTGCAGGAAA	TCCCTCCAATCACAAACGGGATGA
Ccnd1	ATTGGTCTTTCATTGGGCAACGGG	GGCCAATTGGGTTGGGAAAGTCAA
Cdkn1b	AGAAATCTCTTCGGCCCGGTCAAT	TTGCGCTGACTCGCTTCTTCCATA
Crebbp	TTGTGATTCATCTGCATGCTGGGC	ATTTGGAGCGGCGTAAGGAAGAGA
Ep300	AGCACACACAGCTGATCCAGAGAA	CGCTGGCACTTGTGAGCATGTAAA
Foxo1	ACATTTCGTCCTCGAACCAGCTCA	ATTTCAGACAGACTGGGCAGCGTA
Fzd7	GGAACGCATTCACACACACACACA	AACCTCGGATTTCTGTGGCTTTGC
Gapdh	AACATTGGCATTGTGGAAGGGCTC	TGGAAGAGTGGGAGTTGCTGTTGA
Gsk3b	TCCGAGGAGAGCCCAATGTTTCAT	TGGACGTGTAATCAGTGGCTCCAA
Igf1	AAGCAAGAGGCTAGGGATTTGGGA	ATTTGACTGAGGTCACAGGGTGGT
Igfbp3	TGATTTCAGACGTTCCGTGGCTGA	AAGTAGATCAGGCCACTTGCGGAT
Igfbp5	ACGGCGAGCAAACCAAGATAGAGA	TCAGCTTCTTTCTGCGGTCCTTCT
Kat2b	TACCTCTTCACCTGCGTCCACAAA	TCACACCCTGTTCAATACTGGGCT
Mapk14	AAGCTCTTGCGCATGCCTACTTTG	AACAGAAACCAGGTGCTCAGGACT
Mapkapk3	TTTGTGAAAGGTCGCACTTGGCTC	AACCCTCTCTGCTCCAATTCTGCT
Rbpj	CCTTGTTACCAAACAGGTTGCGCT	TGGTGAGGCTCGAACCTGAATGAA
Tgfbr1	TGTCAAAGTCAGTCCGTTGGGTCT	ACTTTGAAAGCCACACAAGCCCTG
Tgfbr2	AGATGGCTCGCTGAACACTACCAA	AGAATCCTGCTGCCTCTGGTCTTT
Tgfbr3	AGCACATTGGAGAGACGACCACTT	AGGCTCATCAGAAAGGCTGATGGT
Wnt4	GCAGATGTGCAAACGGAACCTTGA	ACACCTGCTGAAGAGATGGCGTAT

### Emerin Rescue of *Gsk3b*, *Mapk14* and *Igfbp3* Expression

pEGFP (Clontech) or pEGFP-emerin was electroporated into 2 million emerin-null myogenic progenitors using the Neon transfection system (Invitrogen) with 2 pulses of 1225 volts at a width of 30 milliseconds. The electroporated progenitors were plated in separate 150 mm plates and incubated at 33°C, 10% CO_2_. After 16–48 hours Fluorescence Activated Cell Sorting selected GFP or GFP-emerin expressing cells. On average 120,000 GFP-positive cells were received per electroporation. RNA was isolated and cDNA was generated as previously described. *Gsk3b*, *Mapk14*, *Igfbp3* and *Gapdh* mRNA expression was monitored using qPCR. *Gsk3b*, *Mapk14* and *Igfbp3* levels in GFP or GFP-emerin expressing myogenic progenitors were normalized to *Gapdh* expression. *Gsk3b*, *Mapk14* and *Igfbp3* expression in GFP expressing progenitors is set to one and *Gsk3b*, *Mapk14* and *Igfbp3* expression is plotted as fold-change of GFP-emerin expressing progenitors as compared to GFP expressing progenitors.

### miRNA Target Prediction

Misregulated miRNAs were entered into Microcosm Targets, an online repository of predicted and validated targets of all miRBase-annotated miRNAs. Targets of interest within the Wnt, TGF-β, IGF-1, and Notch pathways were selected based on their involvement in myogenesis or nuclear envelope structure and function.

### Western Blotting

H2K cells were grown under proliferative conditions as described. Wildtype and emerin-null cells were treated with 12.5 ng/ml IGF-1 (Millipore, product #GF138) for 10 minutes. Cells were collected and resuspended in NuPAGE LDS Sample Buffer (Invitrogen, product #NP0007) containing 14 µM β-mercaptoethanol (Sigma, product #M3148) at a concentration of 10^7^ cells/ml. An equal number of wildtype and emerin-null cells was separated by electrophoresis on NuPAGE 4-12% Bis-Tris Gels (Invitrogen, product # NP0323BOX), and transferred to nitrocellulose membranes. Membranes were blocked for 1 hour in 3% BSA in phosphate buffered saline (PBS) containing 0.1% Tween 20 (PBS-T) followed by incubation for 2 hours at room temperature or overnight with the indicated primary antibodies. Antibodies used were against p38 (Cell Signaling, product # 9212), phospho-p38 (Cell Signaling, product # 4511S), Kat2b (Abcam, product # ab12188), GSK3β (Cell signaling, product # 9315), Smad2 (Cell Signaling, product # 3102), Myf5 (Abcam, product # ab69997), and γ-tubulin (Sigma, product # T6557) in 3% BSA in PBS-T. Membranes were washed 5 times in PBS-T and placed in appropriate secondary antibody (Thermo Scientific, goat anti-mouse product # 31430, goat anti-rabbit product # 31460) at a concentration of 1∶10,000 for 2 hours at room temperature. Antibody binding was assayed using ECL chemiluminescence detection reagent (GE healthcare, product # RPN2106V1 and RPN2106V2). Images were scanned and band intensities were determined using Quantity One software (Bio-Rad).

## Supporting Information

Figure S1 **Ingenuity Systems analysis to identify effect of emerin loss on IGF-1 signaling pathway.** The network was generated through the use of IPA (Ingenuity Systems, www.ingenuity.com) on normalized mRNA values. Nodes represent molecules in a pathway, while the biological relationship between nodes is represented by a line (edge). Edges are supported by at least one reference in the Ingenuity Knowledge Base. The intensity of color in a node indicates the degree of up- (red) or down- (green) regulation. Nodes are displayed using shapes that represent the functional class of a gene product (Circle =  Other, Nested Circle =  Group or Complex, Rhombus =  Peptidase, Square =  Cytokine, Triangle =  Kinase, Vertical ellipse =  Transmembrane receptor). Edges are marked with symbols to represent the relationship between nodes (Line only =  Binding only, Flat line =  inhibits, Solid arrow  =  Acts on, Solid arrow with flat line =  inhibits and acts on, Open circle =  leads to, Open arrow =  translocates to).
(TIF)

Figure S2 **Ingenuity Systems analysis to identify effect of emerin loss on Notch signaling pathway.** The network was generated through the use of IPA (Ingenuity Systems, www.ingenuity.com) on normalized mRNA values. Nodes represent molecules in a pathway, while the biological relationship between nodes is represented by a line (edge). Edges are supported by at least one reference in the Ingenuity Knowledge Base. The intensity of color in a node indicates the degree of up- (red) or down- (green) regulation. Nodes are displayed using shapes that represent the functional class of a gene product (Circle =  Other, Nested Circle =  Group or Complex, Rhombus =  Peptidase, Square =  Cytokine, Triangle =  Kinase, Vertical ellipse =  Transmembrane receptor). Edges are marked with symbols to represent the relationship between nodes (Line only =  Binding only, Flat line =  inhibits, Solid arrow  =  Acts on, Solid arrow with flat line =  inhibits and acts on, Open circle =  leads to, Open arrow =  translocates to).
(TIF)

Figure S3 **Ingenuity Systems analysis to identify effect of emerin loss on Wnt/β-catenin signaling pathway.** The network was generated through the use of IPA (Ingenuity Systems, www.ingenuity.com) on normalized mRNA values. Nodes represent molecules in a pathway, while the biological relationship between nodes is represented by a line (edge). Edges are supported by at least one reference in the Ingenuity Knowledge Base. The intensity of color in a node indicates the degree of up- (red) or down- (green) regulation. Nodes are displayed using shapes that represent the functional class of a gene product (Circle =  Other, Nested Circle =  Group or Complex, Rhombus =  Peptidase, Square =  Cytokine, Triangle =  Kinase, Vertical ellipse =  Transmembrane receptor). Edges are marked with symbols to represent the relationship between nodes (Line only =  Binding only, Flat line =  inhibits, Solid arrow  =  Acts on, Solid arrow with flat line =  inhibits and acts on, Open circle =  leads to, Open arrow =  translocates to).
(TIF)

Figure S4 **Ingenuity Systems analysis to identify effect of emerin loss on TGF-β signaling pathway.** The network was generated through the use of IPA (Ingenuity Systems, www.ingenuity.com) on normalized mRNA values. Nodes represent molecules in a pathway, while the biological relationship between nodes is represented by a line (edge). Edges are supported by at least one reference in the Ingenuity Knowledge Base. The intensity of color in a node indicates the degree of up- (red) or down- (green) regulation. Nodes are displayed using shapes that represent the functional class of a gene product (Circle =  Other, Nested Circle =  Group or Complex, Rhombus =  Peptidase, Square =  Cytokine, Triangle =  Kinase, Vertical ellipse =  Transmembrane receptor). Edges are marked with symbols to represent the relationship between nodes (Line only =  Binding only, Flat line =  inhibits, Solid arrow  =  Acts on, Solid arrow with flat line =  inhibits and acts on, Open circle =  leads to, Open arrow =  translocates to).
(TIF)

Figure S5 **Protein expression is similar between primary myoblasts and H2K myoblasts.** A) Whole cell lysate from primary myoblasts and H2K myogenic progenitors were separated by SDS-PAGE and western-blotted for Kat2b, GSK3β, Myf5 and γ-tubulin. B) Densitometry was performed on blots in panel A to measure protein expression. Kat2b, GSK3β and Myf5 densitometry were normalized to γ-tubulin for each sample.
(TIF)
