# Supplementary figures and images for: Loss of Emerin Alters Myogenic Signaling and miRNA Expression in Mouse Myogenic Progenitors
Source: PLoS One. 2012 May 11;7(5):e37262. doi: 10.1371/journal.pone.0037262 (PMC3350500; doi:10.1371/journal.pone.0037262)

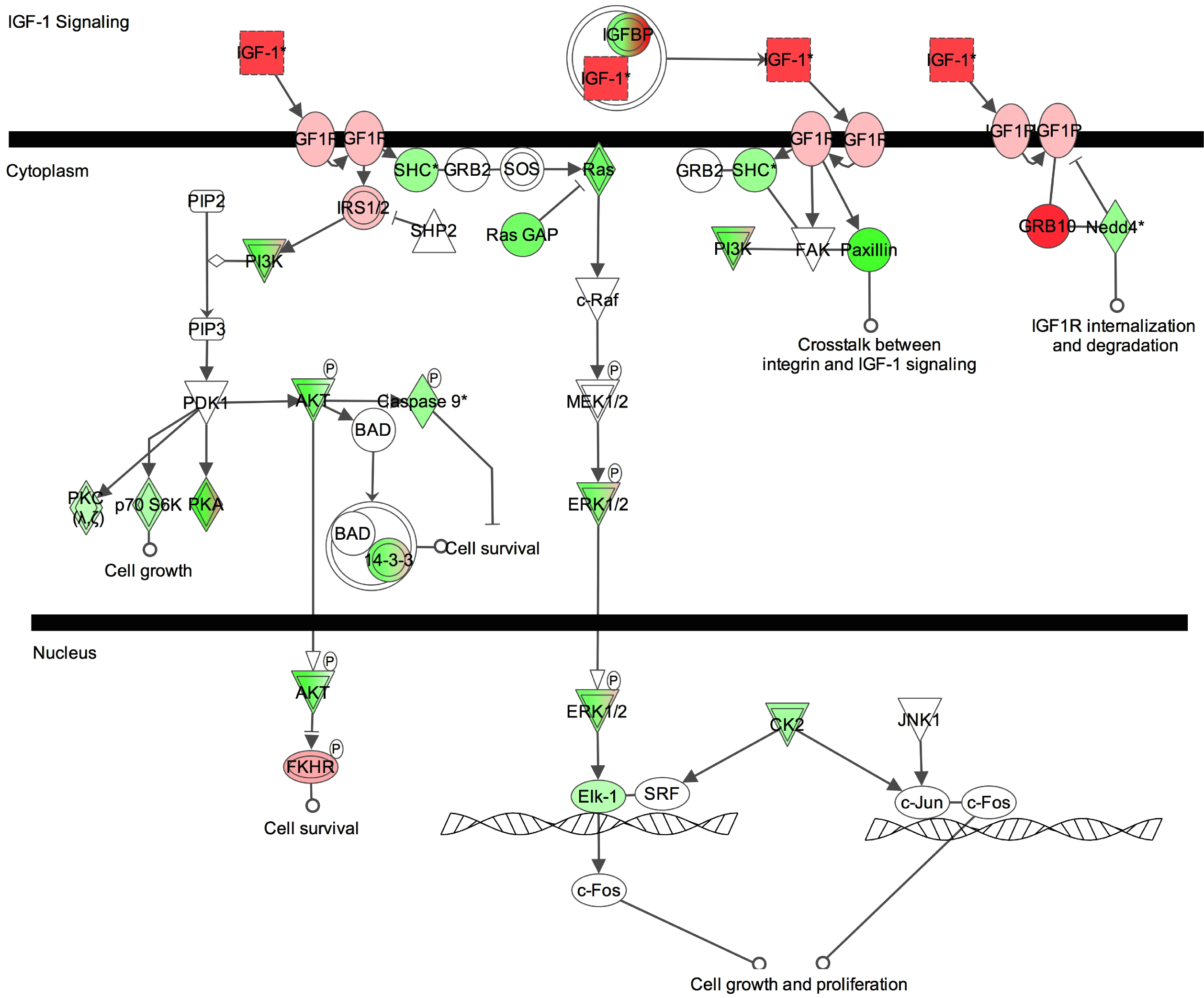

Supplement: Figure S1 — Ingenuity Systems analysis to identify effect of emerin loss on IGF-1 signaling pathway. The network was generated through the use of IPA (Ingenuity Systems, www.ingenuity.com) on normalized mRNA values. Nodes represent molecules in a pathway, while the biological relationship between nodes is represented by a line (edge). Edges are supported by at least one reference in the Ingenuity Knowledge Base. The intensity of color in a node indicates the degree of up- (red) or down- (green) regulation. Nodes are displayed using shapes that represent the functional class of a gene product (Circle = Other, Nested Circle = Group or Complex, Rhombus = Peptidase, Square = Cytokine, Triangle = Kinase, Vertical ellipse = Transmembrane receptor). Edges are marked with symbols to represent the relationship between nodes (Line only = Binding only, Flat line = inhibits, Solid arrow = Acts on, Solid arrow with flat line = inhibits and acts on, Open circle = leads to, Open arrow = translocates to). (TIF) [file pone.0037262.s001.tif]

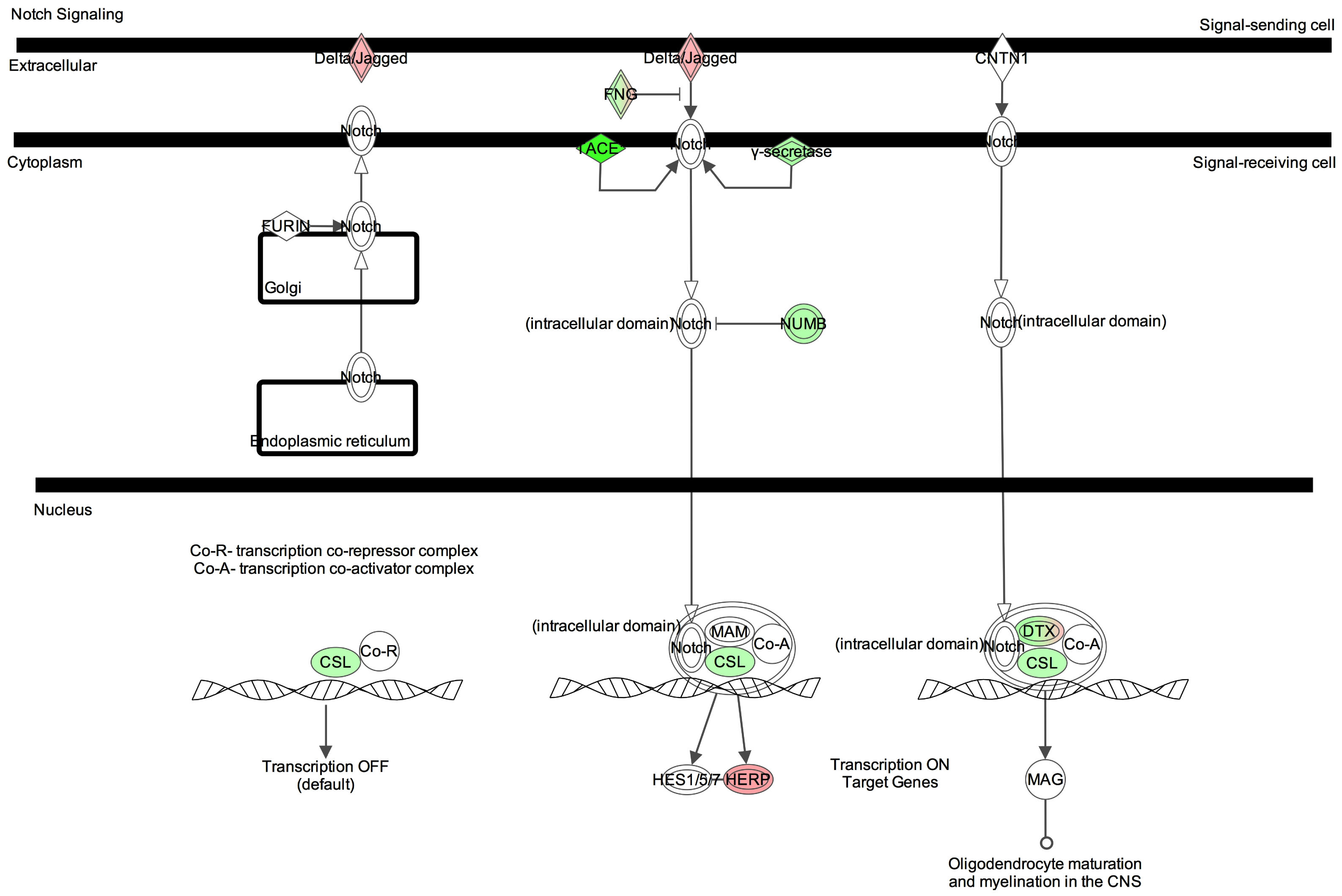

Supplement: Figure S2 — Ingenuity Systems analysis to identify effect of emerin loss on Notch signaling pathway. The network was generated through the use of IPA (Ingenuity Systems, www.ingenuity.com) on normalized mRNA values. Nodes represent molecules in a pathway, while the biological relationship between nodes is represented by a line (edge). Edges are supported by at least one reference in the Ingenuity Knowledge Base. The intensity of color in a node indicates the degree of up- (red) or down- (green) regulation. Nodes are displayed using shapes that represent the functional class of a gene product (Circle = Other, Nested Circle = Group or Complex, Rhombus = Peptidase, Square = Cytokine, Triangle = Kinase, Vertical ellipse = Transmembrane receptor). Edges are marked with symbols to represent the relationship between nodes (Line only = Binding only, Flat line = inhibits, Solid arrow = Acts on, Solid arrow with flat line = inhibits and acts on, Open circle = leads to, Open arrow = translocates to). (TIF) [file pone.0037262.s002.tif]

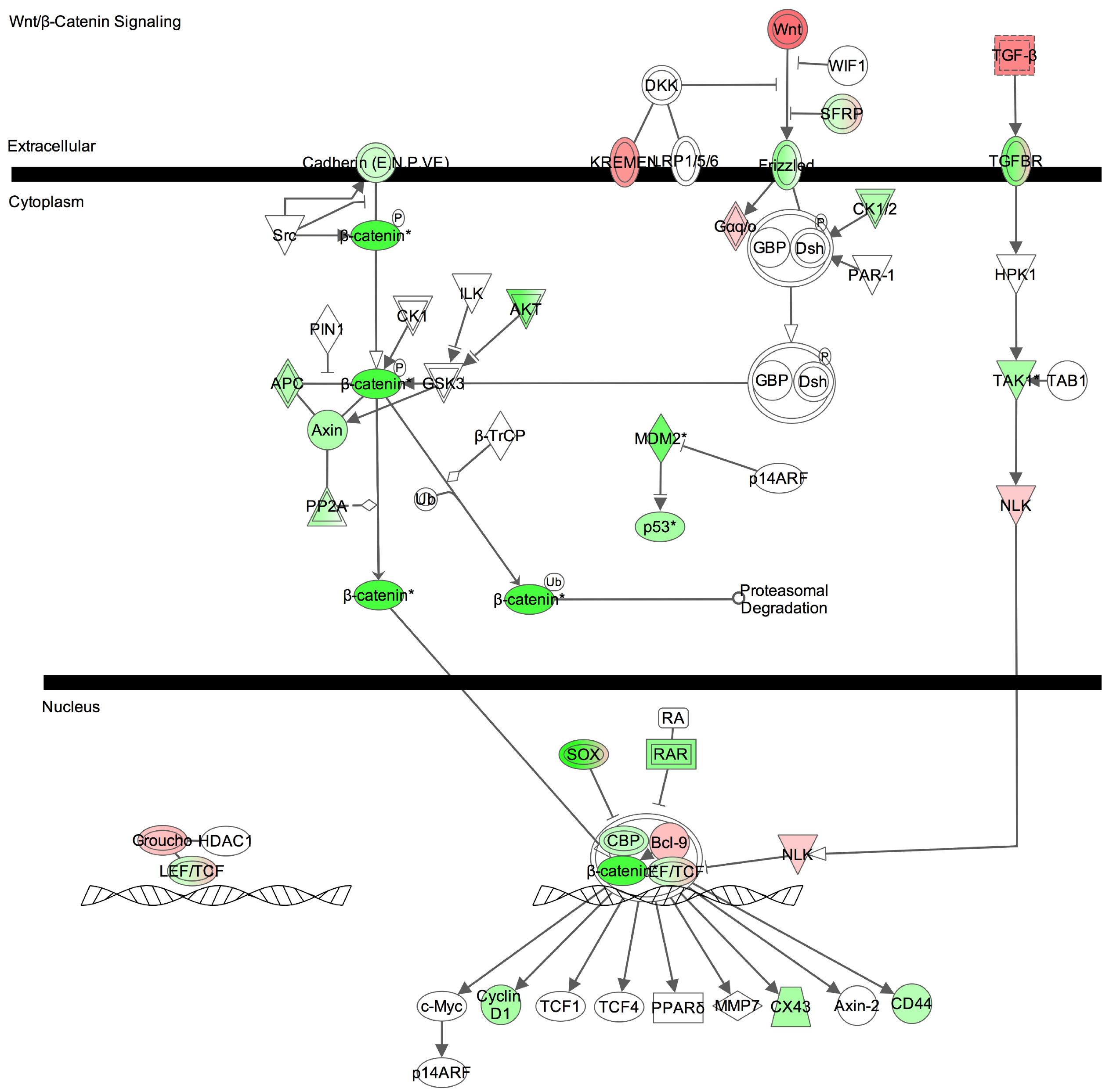

Supplement: Figure S3 — Ingenuity Systems analysis to identify effect of emerin loss on Wnt/β-catenin signaling pathway. The network was generated through the use of IPA (Ingenuity Systems, www.ingenuity.com) on normalized mRNA values. Nodes represent molecules in a pathway, while the biological relationship between nodes is represented by a line (edge). Edges are supported by at least one reference in the Ingenuity Knowledge Base. The intensity of color in a node indicates the degree of up- (red) or down- (green) regulation. Nodes are displayed using shapes that represent the functional class of a gene product (Circle = Other, Nested Circle = Group or Complex, Rhombus = Peptidase, Square = Cytokine, Triangle = Kinase, Vertical ellipse = Transmembrane receptor). Edges are marked with symbols to represent the relationship between nodes (Line only = Binding only, Flat line = inhibits, Solid arrow = Acts on, Solid arrow with flat line = inhibits and acts on, Open circle = leads to, Open arrow = translocates to). (TIF) [file pone.0037262.s003.tif]

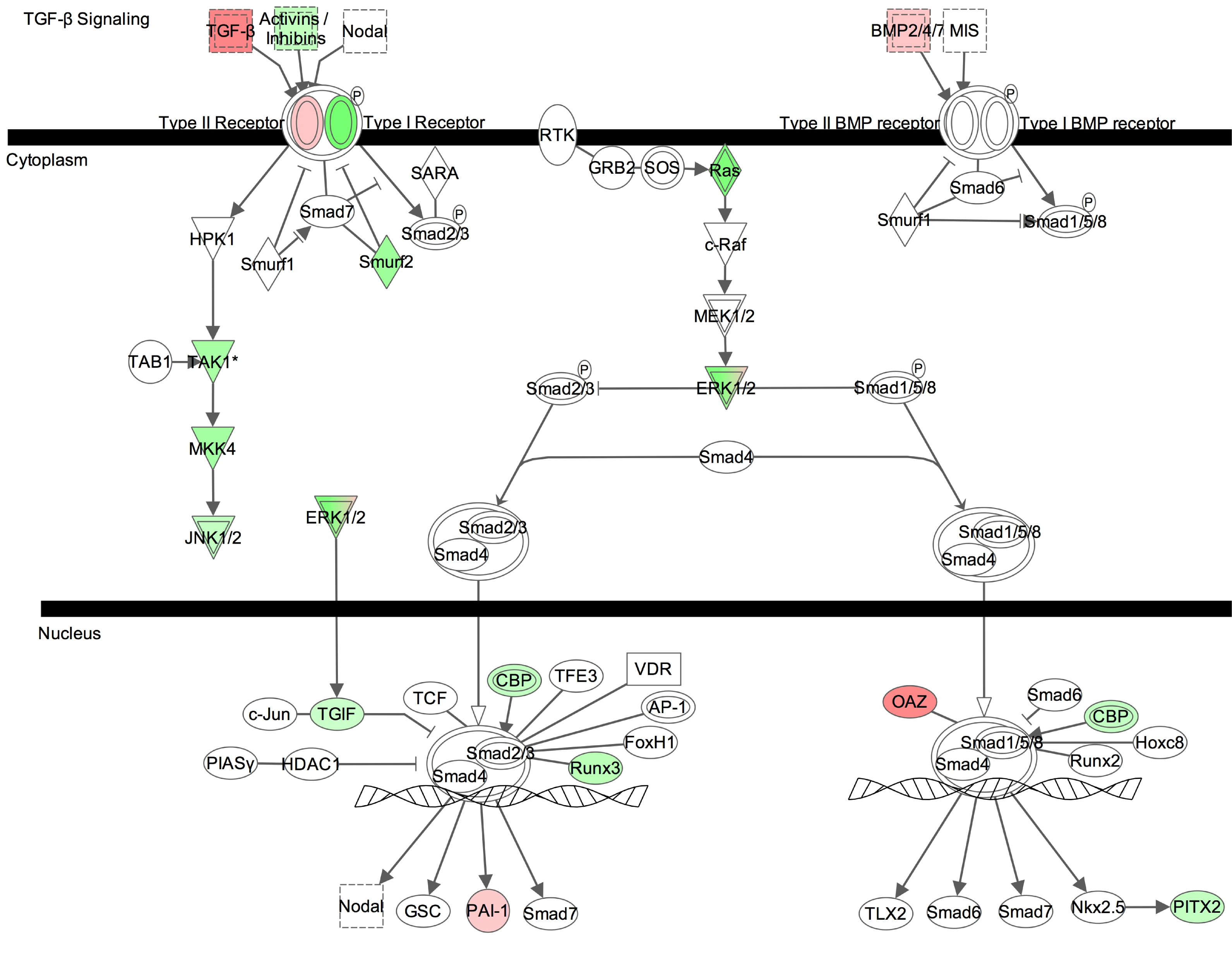

Supplement: Figure S4 — Ingenuity Systems analysis to identify effect of emerin loss on TGF-β signaling pathway. The network was generated through the use of IPA (Ingenuity Systems, www.ingenuity.com) on normalized mRNA values. Nodes represent molecules in a pathway, while the biological relationship between nodes is represented by a line (edge). Edges are supported by at least one reference in the Ingenuity Knowledge Base. The intensity of color in a node indicates the degree of up- (red) or down- (green) regulation. Nodes are displayed using shapes that represent the functional class of a gene product (Circle = Other, Nested Circle = Group or Complex, Rhombus = Peptidase, Square = Cytokine, Triangle = Kinase, Vertical ellipse = Transmembrane receptor). Edges are marked with symbols to represent the relationship between nodes (Line only = Binding only, Flat line = inhibits, Solid arrow = Acts on, Solid arrow with flat line = inhibits and acts on, Open circle = leads to, Open arrow = translocates to). (TIF) [file pone.0037262.s004.tif]

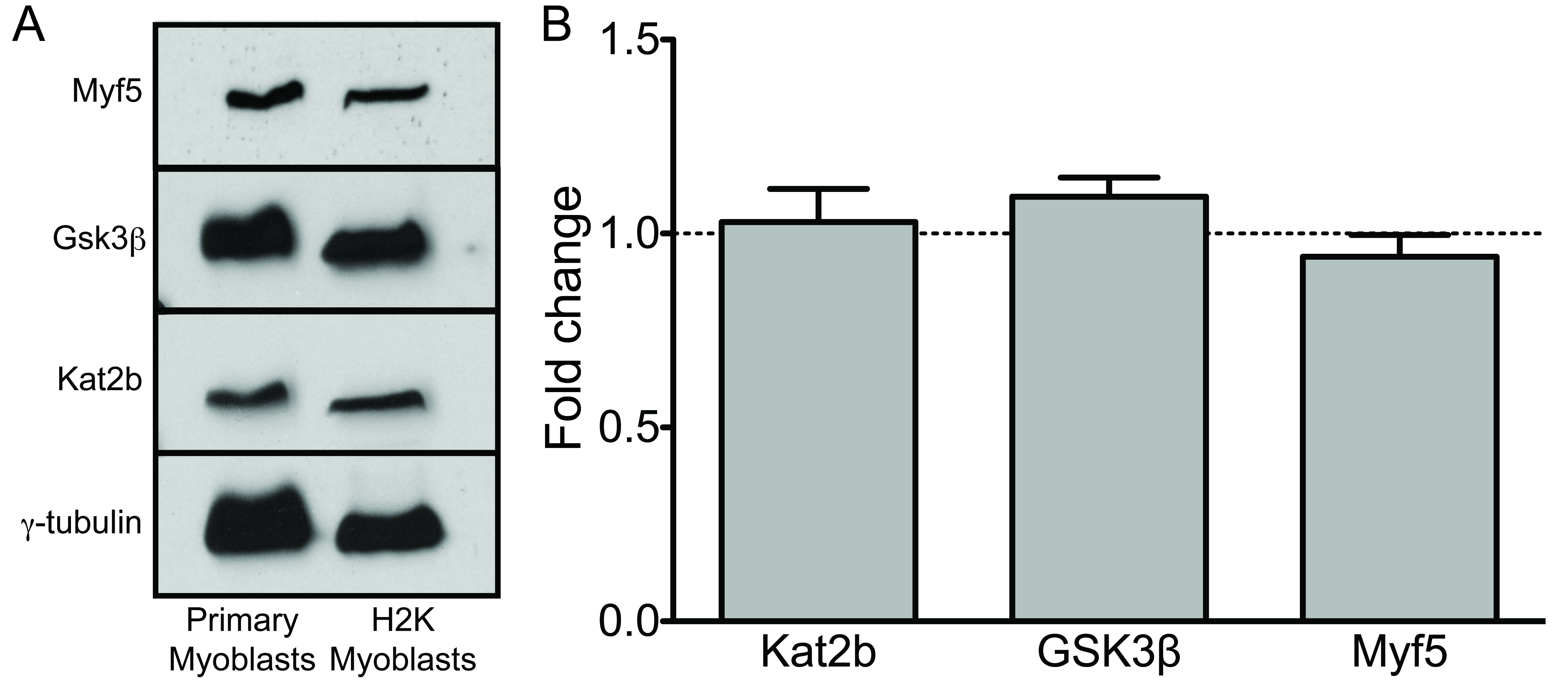

Supplement: Figure S5 — Protein expression is similar between primary myoblasts and H2K myoblasts. A) Whole cell lysate from primary myoblasts and H2K myogenic progenitors were separated by SDS-PAGE and western-blotted for Kat2b, GSK3β, Myf5 and γ-tubulin. B) Densitometry was performed on blots in panel A to measure protein expression. Kat2b, GSK3β and Myf5 densitometry were normalized to γ-tubulin for each sample. (TIF) [file pone.0037262.s005.tif]
